# Mapping Habitats and Developing Baselines in Offshore Marine Reserves with Little Prior Knowledge: A Critical Evaluation of a New Approach

**DOI:** 10.1371/journal.pone.0141051

**Published:** 2015-10-23

**Authors:** Emma Lawrence, Keith R. Hayes, Vanessa L. Lucieer, Scott L. Nichol, Jeffrey M. Dambacher, Nicole A. Hill, Neville Barrett, Johnathan Kool, Justy Siwabessy

**Affiliations:** 1 Digital Productivity Flagship, Commonwealth Scientific and industrial Research Organisation (CSIRO), Brisbane, QLD, Australia; 2 Digital Productivity Flagship, Commonwealth Scientific and industrial Research Organisation (CSIRO), Hobart, TAS, Australia; 3 Institute for Marine and Antarctic Studies, University of Tasmania, Hobart, Tasmania, Australia; 4 Geoscience Australia, Canberra, ACT, Australia; University of Sydney, AUSTRALIA

## Abstract

The recently declared Australian Commonwealth Marine Reserve (CMR) Network covers a total of 3.1 million km^2^ of continental shelf, slope, and abyssal habitat. Managing and conserving the biodiversity values within this network requires knowledge of the physical and biological assets that lie within its boundaries. Unfortunately very little is known about the habitats and biological assemblages of the continental shelf within the network, where diversity is richest and anthropogenic pressures are greatest. Effective management of the CMR estate into the future requires this knowledge gap to be filled efficiently and quantitatively. The challenge is particularly great for the shelf as multibeam echosounder (MBES) mapping, a key tool for identifying and quantifying habitat distribution, is time consuming in shallow depths, so full coverage mapping of the CMR shelf assets is unrealistic in the medium-term. Here we report on the results of a study undertaken in the Flinders Commonwealth Marine Reserve (southeast Australia) designed to test the benefits of two approaches to characterising shelf habitats: (i) MBES mapping of a continuous (~30 km^2^) area selected on the basis of its potential to include a range of seabed habitats that are potentially representative of the wider area, versus; (ii) a novel approach that uses targeted mapping of a greater number of smaller, but spatially balanced, locations using a Generalized Random Tessellation Stratified sample design. We present the first quantitative estimates of habitat type and sessile biological communities on the shelf of the Flinders reserve, the former based on three MBES analysis techniques. We contrast the quality of information that both survey approaches offer in combination with the three MBES analysis methods. The GRTS approach enables design based estimates of habitat types and sessile communities and also identifies potential biodiversity hotspots in the northwest corner of the reserve’s IUCN zone IV, and in locations close to shelf incising canyon heads. Design based estimates of habitats, however, vary substantially depending on the MBES analysis technique, highlighting the challenging nature of the reserve’s low profile reefs, and improvements that are needed when acquiring MBES data for small GRTS locations. We conclude that the two survey approaches are complementary and both have their place in a successful and flexible monitoring strategy; the emphasis on one method over the other should be considered on a case by case basis, taking into account the survey objectives and limitations imposed by the type of vessel, time available, size and location of the region where knowledge is required.

## Introduction

Australia first made a commitment to develop a National Representative System of Marine Protected Areas (NRSMPA) in 1991. This commitment was renewed in 1993, with the ratification of the 1992 Convention on Biodiversity [1]. At the 2002 World Summit on Sustainable Development many nations (including Australia, USA and South Africa) committed to establishing a network of Marine Protected Areas (MPAs) by 2012 [2–3]. The first MPA network under the NRSMPA was formally declared in July 2007 [1]. These MPA networks contain areas with varying levels of protection (International Union for Conservation of Nature (IUCN) categories; [4]). Traditionally, individual MPAs are relatively small, but a growing body of research indicates that larger reserves are better at protecting species, representing biodiversity and sustaining populations [5–8]. As a result, recently designated MPAs have protected increasingly larger areas [9].

In 2012, the Australian federal government established 40 new Commonwealth Marine Reserves (CMRs) [10]. These ‘reserves’ are composed of areas designated 1a (Sanctuary Zone) to VI (General Use Zone) and range in size from around 1,000 to 1,000,000 km^2^, making the latter some of the largest in the world. In total, 60 CMRs and approximately 3.1 million km^2^ of ocean and seafloor are now part of the Australian CMR Network. The marine reserve network was established to protect and maintain marine biodiversity and to help ensure the long-term ecological viability of Australia’s marine ecosystems [11]. Management of this new network, however, is hindered by the absence of prior knowledge on the nature and extent of benthic habitats and assemblages within protected areas, particularly shelf waters where diversity is richest and anthropogenic pressures are greatest. To measure the performance of the CMR network against its stated objectives, and develop and inform management plans, the Australian Government requires the capacity to undertake targeted, cost-effective and sustained data collection. Understanding the nature and distribution of benthic habitats at a fine-scale will assist in this task.

Multibeam echo sounders (MBES) are currently the most efficient acoustic remote sensing tool for fine-scale seabed mapping in depths beyond the visible limit as they provide complete spatial coverage of bathymetry and relative hardness and roughness (via backscatter intensity) of the seabed. It is well established that acoustic returns from the seabed can identify seabed characteristics such as substrate type, grain size distribution, porosity and density [12–18]. While approximately 25% (~1.314 million km^2^) of the continental slope, rise and abyss around the Australian continent has been mapped by multibeam sonar [19], only about 2% (40,424 km^2^) of the Australian shelf has been mapped. The proportion of the Australian CMR network (excluding the Great Barrier Reef (GBR)) that has been mapped varies between reserves and between different IUCN zones within reserves but on average is approximately 12.4%.

It is estimated that that 306,627 km^2^ of the CMR network lies within the biologically diverse 40m to 200m bathome (*pers comm*. Jason Passioura, based on Geoscience Australia’s 2009 Australian Bathymetry and Topography Grid). MBES data is collected as a series of overlapping swaths whose width is approximately 4 times the water depth, at a vessel speed (under good sea state conditions) of about 6 to 7 knots. Hence within the 40m to 200m depth MBES data is acquired at a rate of about 2 to 10 km^2^ per hour. Based on these estimates, it will take between 3.5 and 17.5 years to map this region, assuming vessels can operate 24 hours a day, 365 days a year.

An alternative approach to continuous MBES is to design a survey to collect information at smaller scales but over a broader spatial area and use sampling inference methods to draw conclusions about the entire region of interest. This approach then allows quantitatively- based biological monitoring strategies to commence during the long lead-in time required to acquire continuous MBES data, yet be based on a methodology that allows results to be representative of the CMR surveyed. In such an instance a flexible probabilistic sample design would be suitable [20].

**Fig 1:**
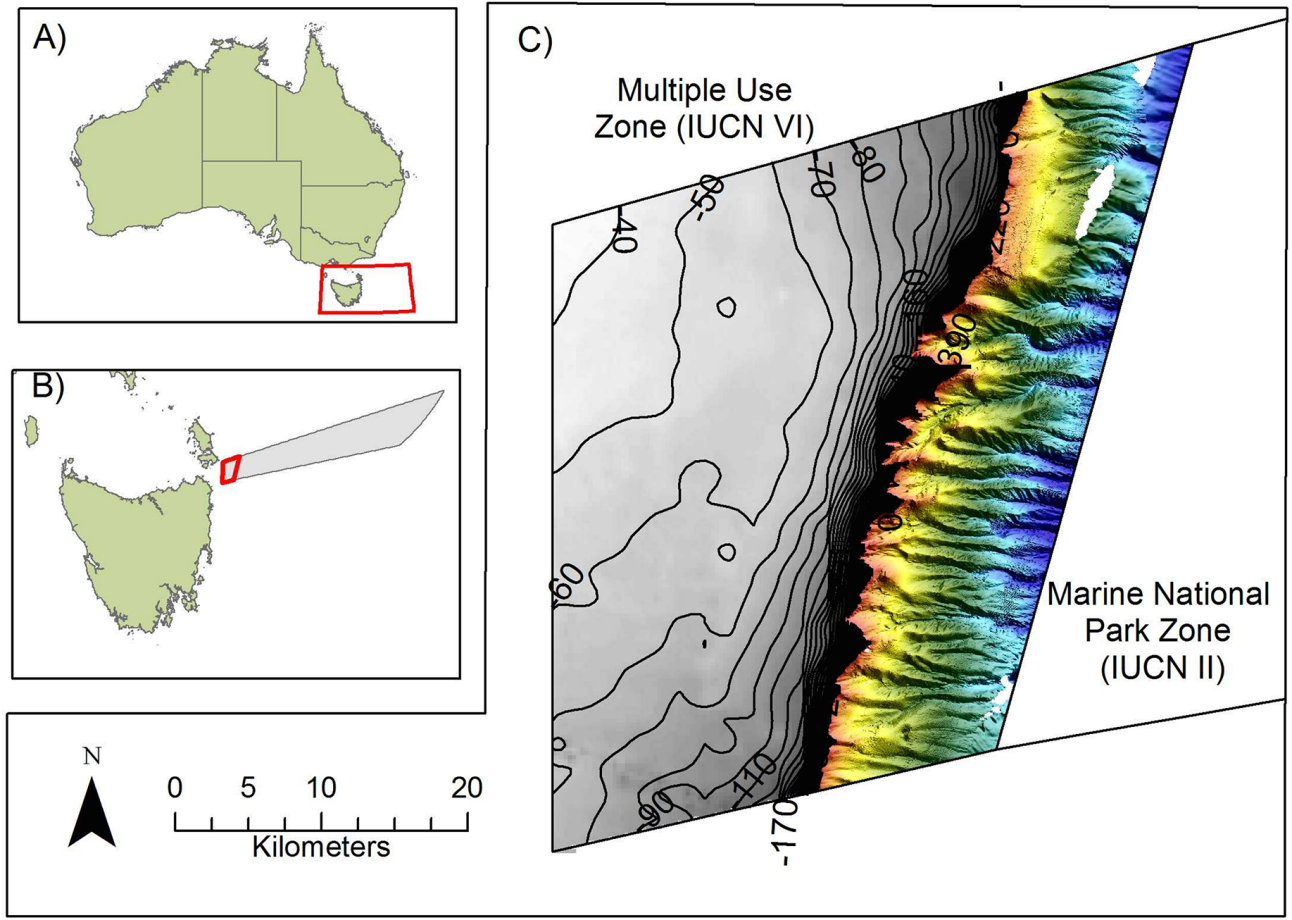
Location of the Flinders Commonwealth Marine Reserve (CMR). A) Australia, with red box highlighting the area of interest shown in B, B) Finders CMR in relation to Tasmania (the area in red is the Multiple Use Zone (IUCN VI), C) Shelf and slope of the Flinders CMR showing the inshore boundaries of the reserve, together with the boundary between IUCN zone VI and zone II, and the extent of existing MBES derived seabed habitat maps, restricted predominately to the slope, prior to the commencement of this study.

To test the viability and performance of such an approach, we conducted a study on the Flinders CMR, a reserve located to the northeast of Tasmania that epitomises the state of benthic mapping on the continental shelf within many of the CMRs. The Flinders CMR extends from 3 to 200 nm offshore and spans a depth range of 40 to 3000 m (Fig 1). The reserve incorporates an area of continental shelf covering 774 km^2^ that is approximately 30 km wide, with the shelf break at 150–160 m water depth. Two shelf-incising canyons occur within the CMR, with heads that extend up to 600 m into the shelf. MBES data (20 m resolution) are available for the entire slope of the CMR (http://www.marine.csiro.au/geoserver/index.html), but the only bathymetric data available for the shelf is very coarse (250 m resolution). A small area (~38 km^2^) of multibeam data was collected at eight sites on the shelf in 2011 as part of a reconnaissance survey to confirm the presence of reef and canyon head systems in the CMR.

We examined two approaches to characterizing the poorly mapped shelf habitats within the Flinders CMR: (i) MBES mapping of a continuous (~30 km^2^) area selected for its potential to include low profile reef and soft sediment habitats that are thought to be representative of the reserve’s shelf habitats, versus; (ii) a flexible probabilistic approach that used targeted mapping of a greater number of smaller, but spatially balanced locations using a Generalized Random Tessellation Stratified (GRTS) sample design.

We describe a broadly applicable method to quantitatively assess the distribution of cross shelf habitats, and their associated biota. We present the first quantitative estimates of the extent of sessile biological communities, together with the area of reef, mixed (reef and soft) and soft sediment habitats on the shelf of the Flinders CMR based on three methods for classifying habitat from MBES data. We use sea bed images from the representative continuous mapped area to ground truth the three habitat classification methods, assess the potential accuracy of our habitat estimates in light of these results, and highlight the challenges that the low profile reefs of the Flinders CMR present in this context. Estimates of the amount and distribution of habitat types and their associated biota, provide the foundation with which to address a range of management objectives.

## Material and Methods

### Sample collection

#### Probabilistic sites

The most common choice of sample design for environmental monitoring programs is either simple random sampling or systematic sampling. While these are simple and commonly understood designs, they have some undesirable properties. Simple random samples often result in the sites being concentrated in some patches, and sparse in others, thereby potentially missing important areas. While systematic sampling overcomes this problem, a direct design-based variance estimator does not exist, preventing an accurate determination of the variances associated with estimated quantities [20]. Generalized Random Tessellation Stratified (GRTS) designs [21] overcome these issues by providing a sample of sites with good spatial balance and a stable and approximately unbiased variance estimator. GRTS was originally developed for sampling streams and stream networks [22] and has since been used in various applications including wetland assessment [23] and monitoring natural resource condition [24]. To the best of our knowledge it has not been previously used for seabed sampling surveys.

We selected a GRTS master sample (an overly large sample), rather than a realistic sample size specific to this study, so that should sampling take place in the future, sample locations would be pre-determined thus avoiding ad hoc sampling on each new occasion. To minimize transit time we chose a larger than feasible sample size from the GRTS master sample, and then identified the shortest route between sites for subsets in increments of five (i.e. 30 sites, 35 sites,….100 sites) using the TSP package in R [25]. The field crew was instructed on how to change from one subset (sample size) to another to improve sampling flexibility while retaining a statistically robust design. The survey started on the 30 site route but was able to supplement this with an extra 10 sites to achieve 40 samples over four eight hour days (including traverses to and from safe overnight anchorages).

The substrate at each of the 40 GRTS locations was determined by examining the footage from a drop camera lowered to the seafloor at each site for approximately one minute. The drop camera was deployed while drifting as close to the centre of the site as possible, and the habitat in the imagery was assigned to one of three categories: hard reef (hard), mixed reef and sand (mixed) or soft sand (soft). One pass of MBES swath was subsequently made over each site resulting in swath sizes between 0.014 km^2^ and 0.058 km^2^. The variability in swath sizes was due to the sampling covering different water depths—transect lengths were the same. To ensure that each GRTS site had equivalent weight in the sample estimates, the estimated area of each substratum within each site was rescaled to an area of 0.04km^2^ using the proportion of soft, mixed and hard substrate derived from the MBES analysis (see below). Appropriate ethics (University of Tasmania Animal Ethics Permit: A12514) and fieldwork (Australian Government Director of National Parks Approval of Research Activities in the Southeast Commonwealth Marine Reserve Network. Ref No 07/10622) approvals were obtained for this work.

Design-based estimates of habitat area were calculated for the MBES data using the *spsurvey* package in the R statistical program [26–27]. Prior to performing the estimation, the survey first-order inclusion probabilities were adjusted to account for the change in the number of sites actually visited compared to those selected (because we purposefully selected more sites than could feasibly be visited). The GRTS design uses the continuous population analog to the Horvitz-Thomspson estimator [28] to produce estimates of population characteristics. The associated local neighborhood variance estimator has been shown to be stable and approximately unbiased under a range of simulation scenarios [22].

In a second-phase (conducted 5 weeks later) samples of the biological communities on the seabed were collected used a towed stereo camera system (TSCS). Photographic samples were collected from two TSCS transects (~200m), intersecting approximately at the centroid of 11 GRTS sites (8 reef and 3 soft) selected from the phase I sites with inclusion probabilities amended to favor mixed reef systems. Ten images from both transects were subsequently selected using GRTS (points along a line) and converted to a standard 1m^2^ area using Transect measure software [29] prior to scoring. The biota or substrate falling underneath twenty-five randomly selected points in each image was then scored to level 6 in the Collaborative and Automated Tools for Analysis of Marine Imagery (CATAMI) classification scheme [30]. Design-based estimates of biota were calculated using the *spsurvey* package, and for ease of presentation were aggregated to CATAMI levels 4 and 5.

#### Judgmental site

A continuously mapped area, covering approximately 30 km^2^, was selected on the basis of reconnaissance completed in 2011. The reconnaissance identified this area to be low profile reef on the outer continental shelf adjacent to two shelf-incising canyon heads. The proximity of reef close to shelf incising canyon heads was deemed to be potentially important, because it captured a range of habitat types but this is not necessarily representative (in the same proportion) of the unsurveyed areas of the CMR.

Habitat classifications produced from the MBES were validated in the continuously mapped patch, using imagery from an Autonomous Underwater Vehicle (AUV). The AUV collects imagery of fixed areal coverage (approximately 1.5m^2^) from a relatively consistent height (2m) above the seabed. The estimated positional accuracy of the geo-located imagery is less than 2m which matches well the resolution of the MBES data. Habitat classifications were not validated in the GRTS component of the study as the patches surveyed were small and the positional accuracy of the towed video system relatively large compared to the features of interest. Within the contiguously mapped area, 24 one kilometer long AUV transects were conducted. These transects targeted reefs (based on the MBES classification) by establishing start points and line spacing using GRTS with an inclusion probability heavily biased towards hard substrate. Thirty images from each transect, sampled using GRTS, were assessed for their dominant and subdominant substrate type using the CATAMI classification scheme (level 3–4 in the physical classification). The combinations of these categories were subsequently simplified to three classes (hard, mixed and soft) using the decision rules in S1 Table, to compare to the MBES classifications.

### Multibeam sonar mapping

#### Collection methodology

Bathymetry and backscatter data were acquired using a *Kongsberg* EM3002(D) 300 kHz multibeam sonar (MBES) system in single transducer mode mounted on the RV *Challenger* in June and July 2012. Both data sets were processed to an established standard using *Caris HIPS/SIPS* v6.1 software and *CMST-GA MB Process* v10.10.17.0 software respectively [31, 32]. The final bathymetry surface was gridded at a resolution of 2 m within the Caris software. The final backscatter surface was created within *CMST-GA MB Process* toolbox at a resolution of 2 m. The processed MBES data were then interpreted using three analysis techniques: digitization (manual mapping), Geographic Object Based Image Analysis (GEOBIA) and algorithms based on angular response curves of acoustic backscatter.

#### Manual mapping of seabed features

Areas of hard, mixed and soft seabed were hand digitised in ArcGIS for the continuous area and 40 GRTS sites. Polygons were drawn using a combination of the 2 m grid of bathymetry, seabed slope and backscatter intensity. The “soft” classification was assigned to areas characterised by flat to gently undulating bathymetry (< 1° slope) and backscatter intensity with uniform spatial structure (approximate range -25 to -30 dB). The “hard” classification was assigned to areas where the bathymetry and slope indicated raised (~1–5 m) features with local gradients of 5 to 15 degrees and consistently high backscatter intensity (e.g. > -25 dB) across an area greater than ~100 m^2^. The “mixed” substrate classification was assigned where bathymetry and slope show raised areas or was a locally irregular seabed (< 1–2 m vertical range) and/or backscatter intensity with an irregular spatial structure and intensity.

#### Geographic Object Based Image Analysis (GEOBIA)

The multibeam data (both bathymetry and backscatter) were classified into the same three classes using a GEOBIA image processing method [33–35]. This study used e-Cognition Developer 8.0 software to implement the analysis methodology detailed in [33]. We also used bathymetric derivatives such as rugosity and slope in the classification procedure to help define the location of “fuzzy boundaries”, where the transition from one habitat to the next occurs gradually over potentially large areas because, for example, the seabed consists of low profile reef that is partially covered by sand.

#### Algorithms based on angular response curves of acoustic backscatter

The probability of presence of hard seabed (p-hard) in the MBES mapped areas was also calculated using a methodology based on the Angular Response Curve (ARC) [33]. A sample of ARCs from areas of the seabed interpreted to be hard substrate (from the classified AUV data) were selected from the continuously mapped area and averaged. For the continuously mapped area 100 samples (consisting of a co-located classified AUV image and MBES backscatter cell) from a total of 720 were used for the ARC training set. The remaining 620 classified samples were then used for validation. This average ARC was then treated as the “reference” ARC for hard seabed on the Flinders CMR shelf GRTS sites. This reference curve was compared to all other ARCs for the survey area using the Kolmogorov-Smirnov goodness of fit to estimate the probability value of the seabed hardness (p-hard). Finally, the Inverse Distance Weighted (IDW) interpolation technique in ArcGIS was used to produce a continuous layer of the p-hard for all 40 GRTS sites and the 30 km^2^ mapped area. For comparison of results between methods the p-hard probabilities were classified into hard (p-hard >90%), mixed (10–90%) and soft (<10%).

### Accuracy assessment of the substrate classification

Error matrices were calculated to assess the overall prediction accuracy, and the producer’s and user’s accuracy [36], for the three classification algorithms using the AUV data from the continuously mapped site. The overall accuracy is calculated as the total number of correctly classified samples (diagonal elements) divided by the total number of samples. The producer’s accuracy is the fraction of correctly classified samples with regard to all points of that ground truth class (CATAMI class). The user’s accuracy is the fraction of correctly classified samples with regard to all samples classified as this class.

The Kappa statistic was used to quantify the predictive performance of the model [37]. Cohen's kappa coefficient is a statistical measure of inter-rater agreement for qualitative (categorical) items and is generally thought to be a more robust measure than simple percent agreement calculation, but criticized as overly conservative. It has a maximum of 1 when agreement is perfect, 0 when agreement is no better than chance, and negative values when agreement is worse than chance. Other values can be interpreted as: <0.20 poor, <0.40 fair, <0.60 moderate, <0.80 good and to 1 very good [38].

## Results

### Habitat distribution and classification

#### Reef distribution

**Fig 2:**
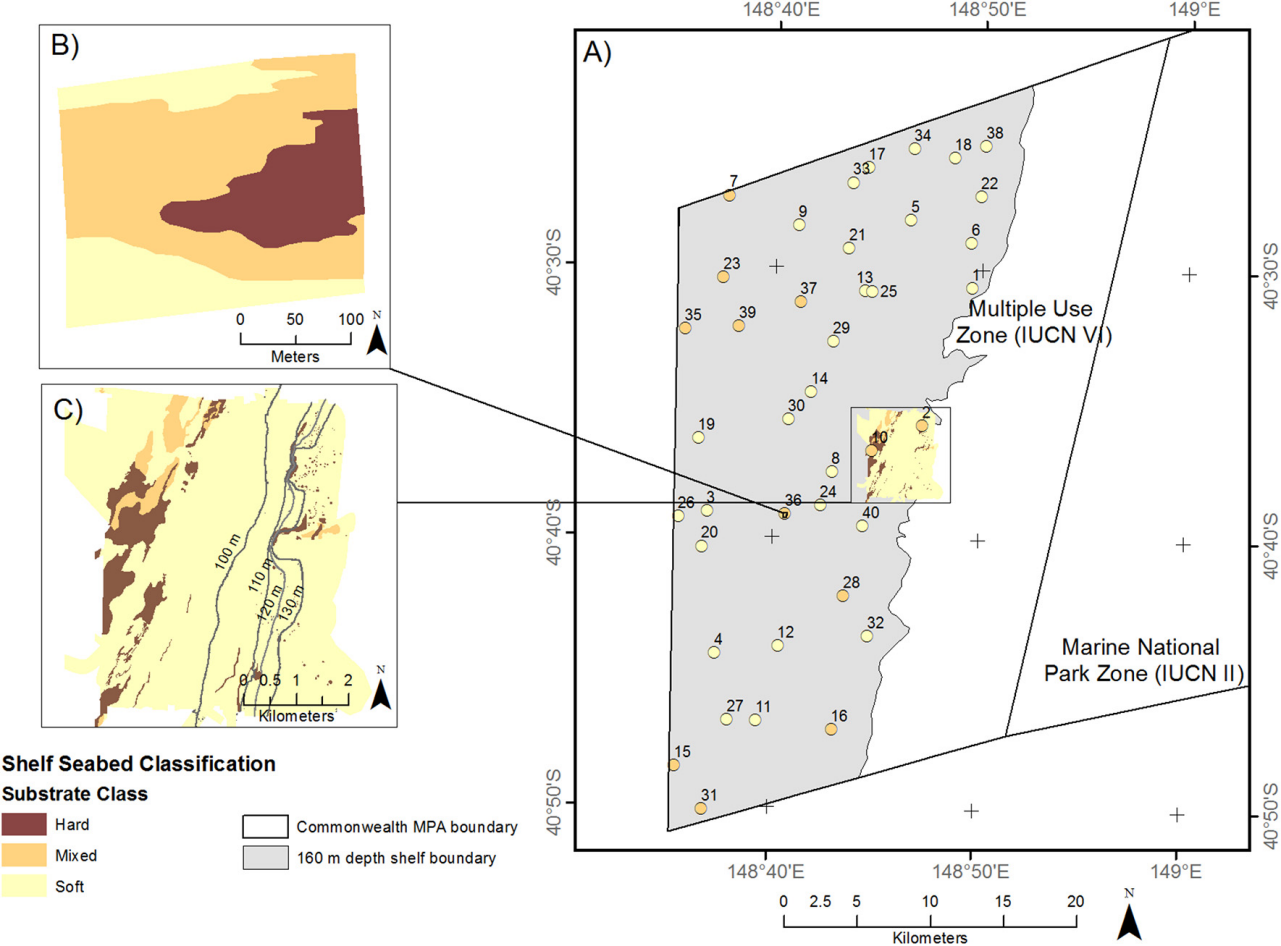
Summary of results of Phase I of the Flinders survey. A) The substratum classification of 40 GRTS sites based on examination of drop camera footage, B) an example of GRTS Site number 36 that has been analysed using GEOBIA, and C) the continuously mapped area classified using GEOBIA.

Examination of the drop camera and MBES data collected at the GRTS sites suggests there is a cluster of mixed reef habitat in the North West corner of the IUCN zone VI region of shelf within the CMR, with reef being patchy otherwise (Fig 2). The bathymetry of the continuously mapped area shows the headwalls of two shelf incising canyons and an area of low profile reef rising 2–3 m above the surrounding sandy seabed.

#### Design-based estimates of substrate for GRTS sites

Across the 40 GRTS sites, our estimates of substrate type differ noticeably across the three analysis methods (Table 1). For hard substrate, the manual interpretation and GEOBIA methods both estimate an average cover of less than 10% across the GRTS sites, but with no overlap in confidence intervals. In contrast, the ARC method yielded an estimate of 43% cover across sites. For soft substrate, GEOBIA and ARC produced similar estimates of cover (46% and 43%, respectively) and with overlapping confidence intervals. Whereas the manual mapping identified an average 77% cover of soft substrate across GRTS sites. Results for mixed substrate are similarly divergent, with manual mapping based on these sites defining about 22% of the substrate across the GRTS sites as mixed, GEOBIA about 44% and ARC 14%.

**Table 1:** The estimated percentage of each substrate across the shelf. The numbers in brackets are the lower and upper 95% confidence intervals based on GRTS survey estimates.

Analysis Method	Hard (%)	Mixed (%)	Soft (%)
***Hand digitised***	1.10 (0.00, 2.22)	21.80 (15.66, 27.93)	77.10 (70.78, 83.42)
***GEOBIA***	9.47 (5.19, 13.75)	44.19 (34.78, 53.61)	46.34 (37.74, 54.93)
***ARC***	43.26 (36.72, 49.79)	13.76 (7.41, 20.10)	42.99 (36.46, 49.51)

#### Estimates of substrate for continuously mapped area

**Fig 3:**
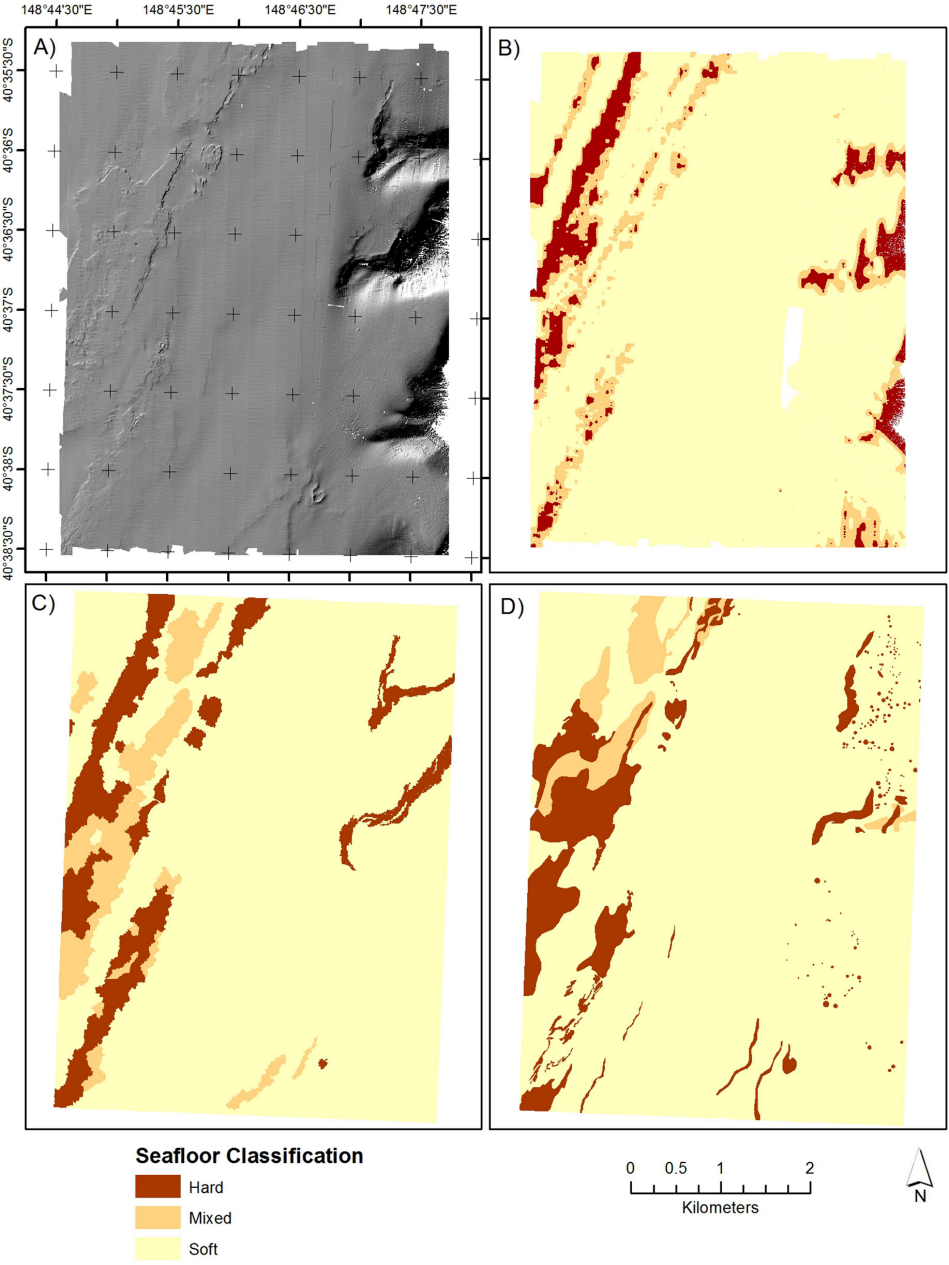
MBES data over continuously mapped area. (A) Hillshade of the bathymetry, (B) probability of hardness derived from the ARC analysis, (C) classification of substrate using GEOBIA, (D) classification of substrate using hand digitization.

Estimates of substrate coverage for the 30 km^2^ mapped area are broadly similar for each of the mapping methods (Table 2). Thus, all three methods show that soft substrate is dominant, covering 83–90% of the mapped area and hard substrate less than 10%. Note that since the continuously mapped area was selected judgmentally there is no variance and hence no confidence interval associated with these estimates. In detail, the substrate maps for each method show variations in the spatial structure of each but general coincidence in the location of hard and soft substrate areas (Fig 3).

**Table 2:** The estimated percentage of each substrate across the continuously mapped area.

Analysis Method	Hard (%)	Mixed (%)	Soft (%)
***Hand digitised***	0.32	16.47	83.21
***GEOBIA***	9.02	3.65	87.33
***ARC***	5.5	4.1	90.4

### Seabed classification accuracy

The accuracy assessment of hand digitised classification of the multibeam data based on the comparison with AUV imagery in the continuously mapped area produced fair agreement with an overall accuracy of 67.64% and Kappa of 0.322 (S2 Table). The assessment of the GEOBIA classification was slightly worse but still fair agreement (overall accuracy was 63.19% %, Kappa of 0.314). The ARC classification of the multibeam data based on the AUV data produced the best agreement (overall accuracy was 74.84%, Kappa of 0.322).

### Sessile biological communities

**Fig 4:**
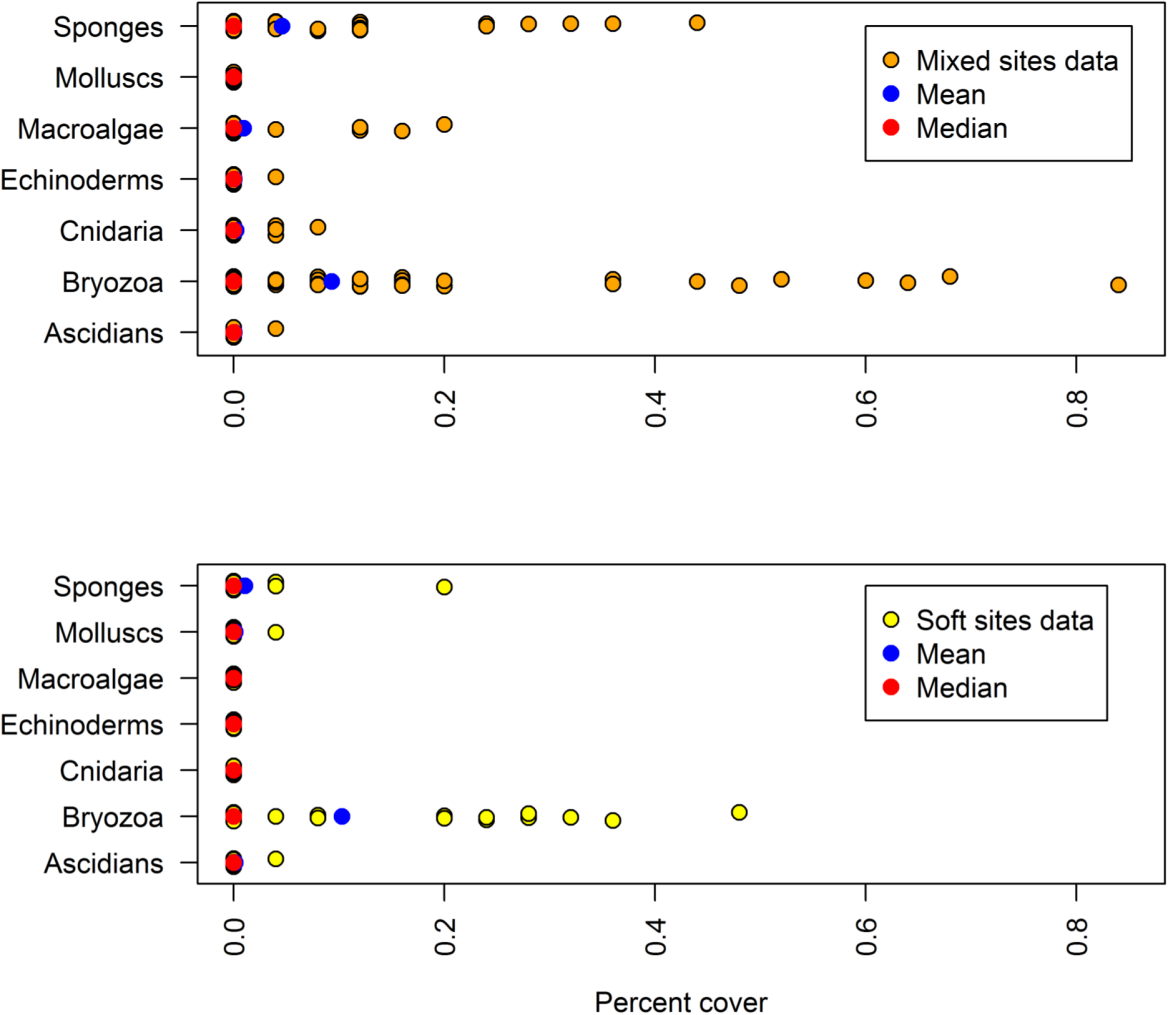
Strip chart of percentage cover of sessile biological communities (grouped to CATAMI level 4) from 10 images from each of the 11 GRTS cells, broken down by mixed and sand habitat.

The seabed communities sampled by the TSCS reflect the patchy, discontinuous distribution of the shelf reef systems. The TSCS imagery contains a large proportion of zero percentage cover (Fig 4), despite choosing the phase II sample inclusion probabilities to be biased towards mixed habitats. Overall, when biological communities are present in the image samples, they tend to be dominated by bryozoa, sponges and to a lesser extent macroalgae, and this is subsequently reflected in the design-based estimates of their total extent on the shelf within the CMR boundaries (Table 3). As expected, percent cover is generally higher in mixed sites (Fig 4), but the presence of groups such as bryozoa and sponges in sites classified as “soft” suggests that small (sub-GRTS cell) scale reefs and/or low profile reefs inundated by a sand veneer were present within these sites.

**Table 3:** The estimated total area (kms^2^) of seabed communities across the Flinders CMR shelf grouped to CATAMI level 4. The numbers in brackets are the lower and upper 95% confidence intervals based on GRTS survey estimates.

Biological group	Total area (kms^2^)
**Ascidians**	0.84 (0.00, 2.04)
**Bryozoa**	77.22 (0.00, 159.3)
**Cnidaria**	0.58 (0.15, 1.01)
**Echinoderms**	0.12 (0.00, 0.32)
**Macroalgae**	2.21 (0.00, 5.85)
**Molluscs**	0.72 (0.00, 1.95)
**Sponges**	16.46 (3.27, 29.65)

**Fig 5:**
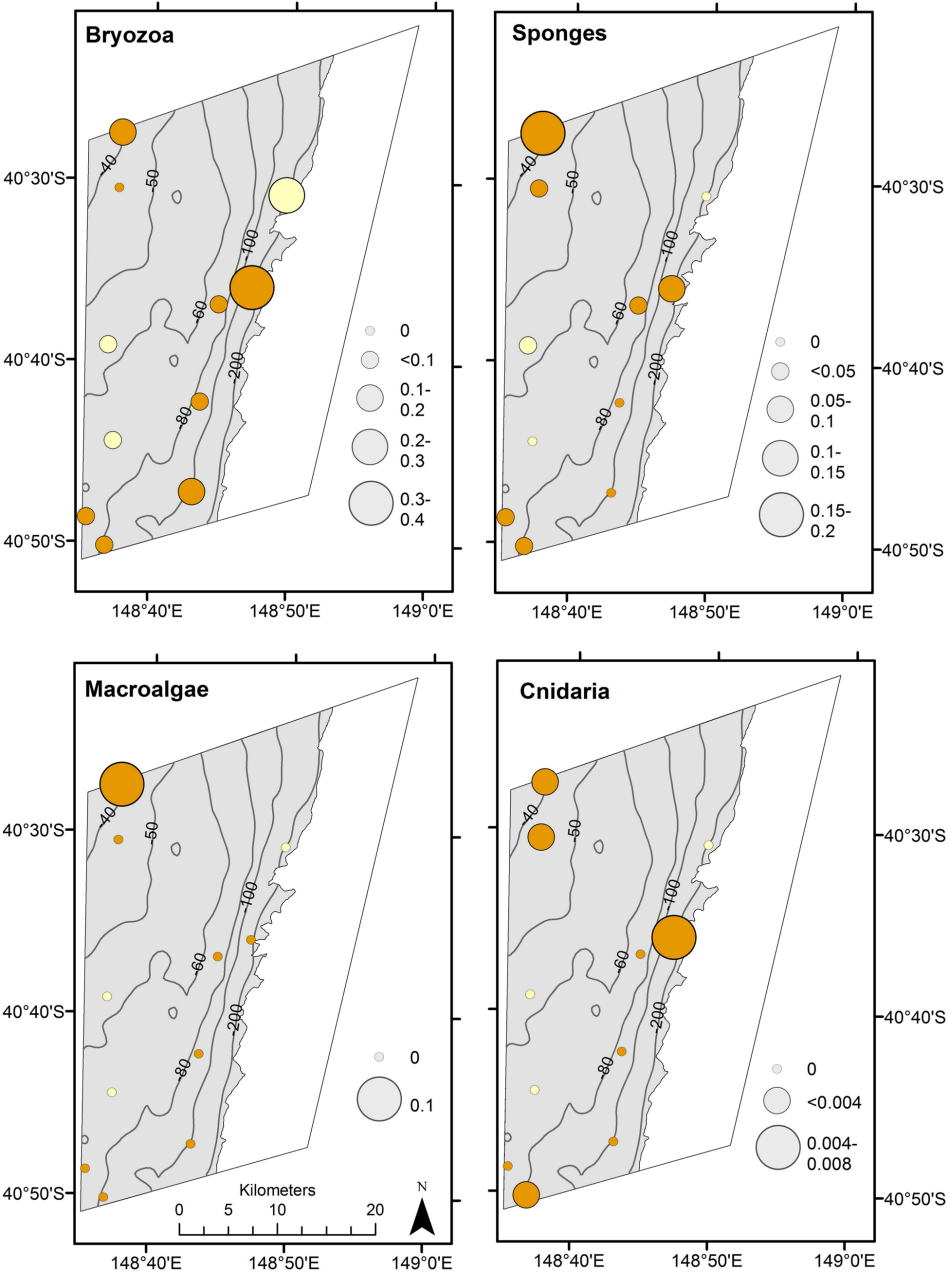
Distribution of percentage cover of sessile biological communities (grouped to CATAMI level 4) at each of the 11 GRTS cells. Orange bubbles represent mixed habitat and yellow bubbles represent sand habitat.

The seabed communities across the shelf of the CMR also show distinct geographical features. Bryozoa appear to be relatively well distributed across the shelf, whereas, macroalgae are clearly restricted to the shallow (< 50m) mixed reef habitat in the north west corner of the IUCN zone VI. This area also appears to be a hotspot for sponges, and to a lesser extent cnidaria (Fig 5). Further examination of the drop camera footage from this area, and the distribution of biota at CATAMI level 5 (S1 Fig), indicate that the seabed communities contain a relatively high proportion of tall biogenic structures with flexible forms such as soft corals, and erect and massive sponges. This suggests that this area of the shelf may experience relatively high currents that keep the reefs free of sand and support diverse filter feeding communities.

The site at the edge of the continental shelf closest to a shelf-incising canyonhead also appears to be a biologically important location (Fig 5). This location displays a relatively high proportion of bryozoa and cnidaria but is clearly too deep for macroalgae. It also appears to be an important location for soft corals, hydroids, massive sponges and unstalked ascidians (S1 Fig).

## Discussion and Conclusions

### Summarizing observations from the survey

The continental shelf within Flinders CMR is dominated by sandy seabed with the remainder of the benthic substrate comprised of sand-inundated low profile reef, where sand forms a thin (millimeter to decimetre scale) veneer over flat bedrock. The reefs are mostly flat features, comprising a series of slightly dipping layers of sedimentary rock that occasionally outcrop and where eroded form a 1–2 m high scarp. In the continuous mapped area, these scarps can be seen to extend ~5 km north-south as a semi-continuous feature, providing a stable rock surface for epibenthic communities of sponges and soft corals to attach. In places, the sand deposits form fields of active bedforms (sand waves, ripples) with sediment transport likely driven by a combination of strong tidal currents that sweep across the shelf and wave-generated currents, particularly during storms. We thus consider the sand veneer on reef to be ephemeral and expect that the boundaries between mapped areas of the different substrate types have the potential to shift, such that small areas of reef may be covered and uncovered over time.

The seabed communities sampled also confirm the patchy and discontinuous distribution of the reef on the Flinders shelf. The biological communities are dominated by bryozoa and sponges and to a lesser extent cnidaria. Macroalgae are only present on the shallow mixed habitats in the northwest of the CMR, which also appears to be a previously undiscovered hotspot for sponges. The site at the edge of the continental shelf closest to a shelf-incising canyonhead is also a biologically important area with relatively high proportions of bryozoans, sponges and cnidarians.

### Substrate mapping methods and accuracy

Low profile, sand-inundated reef habitats are difficult to map. With sand-inundation of reefs ranging from zero at reef high points, through millimetre to decimetre thick patches, to continuous sediment cover, there are often no sharp boundaries for habitats to be differentiated (either by acoustic methods or by visually based methods used for validation). Once rock is covered by a thin veneer of sediment it usually appears identical to thick sediment in visual imagery, with the only clue being a coverage of attached invertebrates that may have settled and grown when the bedrock was exposed at some stage.

Our varying estimates of area of seabed covered by soft, mixed and hard substrates based on manual and automated approaches to analyzing MBES outputs, highlight the challenges when using remote sensing technologies, such as MBES and video, in regions such as the Flinders CMR shelf. Using visual imagery as validation, would, in most instances of reef versus sand habitat prove to be a robust validation method. However, in this environment what is acoustically identified versus what is visually identified is ambiguous (see [39] for further information).

Whilst visual imagery from AUV platforms provides an excellent source of validation due to its high spatial accuracy, number of images along a transect and its ability for accurate repeat surveys, it does have limitations in flat, sandy veneer covered bedrock systems. A clear recommendation for acoustic validation in these low profile sediment dominated systems would be a high resolution sub bottom profiler and/or sediment core surveys that are able to discriminate the overlying sediment from the underlying bedrock.

These challenges are unlikely to be unique to the Flinders CMR, and are no doubt typical of many offshore marine environments where low-lying reefs are partially inundated with sediment, for example drowned relict coastline in the Freycinet region of eastern Tasmania [40]. While MBES derived maps clearly indicate that such features are present, the difficulty arises when their locations, and quantitative measures of their extent are to be delineated. In our study, the relatively small area of the individual GRTS sites, coupled with the characteristically low profile reefs within these sites, made quantification of the mapping process particularly difficult and some of the challenges for accurate quantification of habitats were hard to overcome.

There is by necessity, a level of subjectivity within each of the various mapping methods. The manual method clearly introduces the greatest level of subjectivity in terms of decision-making, and is open to inconsistencies, though efforts to minimise these were made by applying mapping criteria (as described earlier) and quality checks (QC) of results at various stages in the analysis. Regardless, there will always be a degree of uncertainty with the results from manual mapping, the level of which is greater for areas of seabed characterised by subtle spatial variations in substrate type, such as in the Flinders CMR.

The automated GEOBIA and ARC habitat categorisations attempt to overcome such subjectivity, but are still subject to decision rules relating to cut-off points in categories chosen (ARC method), that may, or may not be appropriate, and are influenced by the spatial derivates from a neighboring pixels (GEOBIA) that may swamp the signal of a small scale feature. Accurately defining the cut-off point when veneer over reef becomes “sediment” is difficult to automate and validate with any of these methods. Conversely, areas where sand deposits are hard packed due to the winnowing effects of waves and currents may be modelled as hard seabed by backscatter interpretation methods like ARC due to the strong acoustic return from the hard sand surface. Clearly, these effects will contribute to differences in the detail of the spatial structure of substrate classes identified across the three methods. In addition, for the GRTS sites, the quality of acoustic data was compromised by the acquisition of data on a single pass only (i.e. no replication to detect errors or fill gaps) and rough sea state (that can contribute to missed returns and gaps in the data) such that discriminating between substrate types with indistinct boundaries proved difficult. This may account for some of the differences in habitat estimates between the three analysis methods (Table 1), and help explain why the difference between estimates was much less in the continuously mapped area (Table 2). Reducing these uncertainties in the ARC results would require acquiring more MBES data at each GRTS site, which could be achieved by a second pass or by increasing the size of the GRTS cell, and through employing additional geophysical methods for validation, such as acoustic sub-bottom profiling to accurately infer sediment thickness above the bedrock where it occurs.

### Sampling strategy–continuous mapping versus spatially balanced

A complete inventory of a marine reserve requires that the whole reserve is continuously mapped with high resolution MBES methods. We believe that this should remain the ultimate goal of any management agency. The question, however, is whether we can attain an adequate baseline for quantifying the extent of key physical and biological assets, detecting trends and monitoring changes on the way to achieving this goal, or should we wait for an entire reserve or management zone to be mapped before attempting to establish such a baseline? Clearly, if management plans are to be developed in the short-term in the absence of knowledge, then the answer is the former.

There are two significant advantages of the GRTS approach for initial rapid quantitative knowledge gathering. Firstly, it enables design-based estimates of the area of habitat types (and their associated communities) to be made, with specified confidence intervals. This is important in a long-term monitoring program because trends in these estimates of extent, following repeat surveys, would signal change. Furthermore, because the samples are spatially balanced across the survey area they also tend to span all of the environmental gradients in the survey area, which assists when developing model-based estimates of habitats and communities. Secondly, with enough samples, spatial patterns in the distribution of habitats and key biota become evident and these can be targeted for subsequent sampling or continuous mapping.

The disadvantages of the GRTS approach are the additional transit time between sites and the analysis difficulties associated with the relatively small GRTS cells. The former can be mitigated to some extent by choosing efficient routes between achievable sub-sets of the sample, and our experience suggests that the latter may be addressed by choosing GRTS cells that are larger than 200m^2^, possibly acquired over two passes, and ideally augmented by a sub-bottom profiler. This is particularly true for challenging habitats such as low profile reefs with extensive sand veneer. The analysis of the GRTS cells and the additional transit time, also entail an additional overhead cost, so it is important to emphasize that the advantages that the GRTS-based statistical design offers, do not come without some additional cost. While transect data can be collected while travelling between sample sites, this data does not have the desirable statistical properties of a spatially balanced design and so could be used to provide information about the study area but not the desirable unbiased estimates we have calculated.

The two approaches used in this study, however, are complementary and need not be mutually exclusive. The spatially balanced strategy of GRTS produced estimates (and variances) across the entire 774 km^2^ shelf area of the CMR, hence we know the likely distribution and extent of key benthic physical and biological assets (albeit with some uncertainty), knowledge essential to management planning. In contrast, the continuous mapping provided a high spatial resolution bathymetric map that better informed our understanding of the geomorphic character of a discrete area of the Flinders shelf, and associated biological monitoring at the whole of reef scale. Both approaches have their place in a successful and flexible monitoring strategy, and informing our knowledge of the geomorphology that defines such systems. For example, we used the results from the GRTS-based mapping to design a subsequent survey targeting areas of hard substrate to deploy baited underwater video (BUV) and stereo towed video to document and quantify demersal fish communities in the Flinders CMR as a baseline for future monitoring [41]. The GRTS design also identified potential biodiversity hot spots, such as the north west corner of the CMR and its shelf incising canyon heads, that may be targeted for future continuous mapping if understanding reef communities is an important management priority.

Any future MBES mapping in the Flinders CMR as part of a monitoring program would ideally continue GRTS-based sampling to refine estimates of overall habitat distribution, while continuing to expand the extent of continuous coverage, building upon the existing 30 km^2^ area, and prioritising additional areas based on knowledge obtained from GRTS-based sampling.

In terms of cost-benefit of the two sampling approaches, the larger the region for which little to no prior information exists the greater the potential benefit of the GRTS approach; multibeam mapping large areas can be cost-prohibitive, particularly for the continental shelf. A larger number of sites would be achievable for a CMR of equivalent size if it were closer to sheltered waters and/or with a vessel that is able to map continuously overnight and sample during daylight hours. Even for cases such as the Flinders CMR where a continuously mapped portion of the shelf now exists, adding to this over time will improve coverage (and knowledge) for that site but estimates of substrate coverage would remain biased until most, or all, of the CMR was mapped. Whereas, addition of new GRTS sites over time will maintain unbiased estimates of substrate cover. Additionally, the GRTS approach can be built into a ‘rotating panel’ design [42] so that some sites are visited on a regular basis (every survey), building up a temporal trend to support monitoring, while new sites can be added and sampled less regularly to support discovery while improving the estimate of spatial variation. Both of these aspects are critical in an effective long-term monitoring regime. Clearly, there are trade-offs with each approach and we recommend that the emphasis on one method over the other is considered on a case by case basis taking into account the survey objectives, and limitations imposed by the type of vessel, time available, size and location of the region where knowledge is required.

Ultimately, managers need to choose between developing complete and continuous high-resolution habitat maps of marine reserves at higher cost and resources, or starting to monitor impacts over a shorter time period. If upon reflection, the time and cost of acquiring continuous coverage information suggests that effective management decisions may be delayed for too many years, then a complementary strategy based on a spatially balanced sampling design should be considered. This approach can provide a robust baseline, within a year, and can be used to direct and prioritise subsequent continuous mapping programs.

## Supporting Information

S1 Data Fine scale scoring of the Towed stereo Camera System Data for 11 GRTS sites. (CSV)

S2 Data Area (km^2^) of hard, mixed and sand for three MBES analysis methods for 40 GRTS sites. (CSV)

S3 Data Broad scale scoring of AUV data for Flinders shelf continuous patch. (XLSX)

S1 Fig Distribution of seabed communities grouped to CATAMI level 5. Orange bubbles represent mixed habitat and yellow represent soft habitat.
(DOCX)

S1 Table Mapping of Broadscale AUV imagery categories to MBES crude habitat classification (hard, mixed, soft) used in the MBES accuracy assessments. (DOCX)

S2 Table Accuracy assessment of the hand digitised, GEOBIA and ARC, classification of multibeam and AUV classified points. (DOCX)

## References

[1] Williams A, Bax NJ, Kloser RJ, Althaus F, Barker B, Keith G. Australia's deep-water reserve network: implications of false homogeneity for classifying abiotic surrogates of biodiversity. ICES J Mar Sci. 2009; 66: 214–224.

[2] UNEP-WCMC. National and Regional Networks of Marine Protected Areas: A Review of Progress. UNEP-WCMC, Cambridge 2008.

[3] ANZECC TFMPA. Guidelines for Establishing the National Representative System of Marine Protected Areas. 1998.

[4] Day J, Dudley N, Hockings M, Holmes G, Laffoley D, Stolten S, et al Guidelines for applying the IUCN Protected Area Management Categories to Marine Protected Areas. Gland, Switzerland: IUCN 2012.

[5] Halpern BS, Warner RR. Matching marine reserve design to reserve objectives. Proc Biol. Sci. 2003; 270: 1871–1878. 14561299 10.1098/rspb.2003.2405 PMC1691459

[6] Claudet J, Osenberg CW, Benedetti-Cecchi L, Domenci P, Garcia-Charton J-A, Perez-Ruzafa A, et al Marine reserves: Size and Age do matter. Ecol Lett. 2008; 11: 481–489. 10.1111/j.1461-0248.2008.01166.x 18294212

[7] Gaines SD, White C, Carr MH, Palumbi SR. Designing marine reserve networks for both conservation and fisheries management. Proc. Natl. Acad. Sci. USA. 2010; 107: 18286–18293. 10.1073/pnas.0906473107 20200311 PMC2972919

[8] Edgar GJ, Stuart-Smith RD, Willis TJ, Kininmonth S, Baker SC, Banks S, et al Global conservation outcomes depend on marine protected areas with five key features. Nature. 2014; 506: 216–220. 10.1038/nature13022 24499817

[9] Spalding MD, Meliane IN, Milam A, Fitzgerald C, Hale LZ. Protecting marine spaces: Global targets and changing approaches. Ocean Yearbook Online. 2012; 27: 213–248.

[10] DOTE. Commonwealth Marine reserves. 2014. Available: http://www.environment.gov.au/topics/marine/marine-reserves

[11] Director of National Parks. South-east Commonwealth Marine Reserves Network management plan 2013–23. In: Parks DoN, editor. Canberra. 2013.

[12] Urich RJ. Principles of Underwater Sound. New York, McGraw-Hill; 1983.

[13] Chivers RC, Emerson N, Burns DR. New acoustic processing for underway surveying, Hydro. J. 1990; 56: 9–17.

[14] Collins W, Gregory R, Anderson J. A digital approach to seabed classification, Sea Tech., 8 1996; 83–87.

[15] Bax N, Kloser R, Williams A, Gowlett-Holmes K, Ryan T. Seafloor habitat definition for spatial management in fisheries: a case study on the continental shelf of southeast Australia. Oceanologica Acta. 1999; 22: 705–719.

[16] Lurton X. An Introduction to Underwater Acoustics: Principles and Applications. Springer, Chichester; 2002.

[17] Brown CJ, Blondel P. Developments in the application of multibeam sonar backscatter for seafloor habitat mapping. Applied Acoustics. 2009; 70 (10):1242–1247.

[18] Hamilton LJ. Acoustic seabed segmentation for echosounders through direct statistical clustering of seabed echoes. Continental Shelf Research. 2009; 31:2000–2011.

[19] Kloser RJ and Keith G. Seabed multi-beam back scatter mapping of the Australian continental margin. Acoust Aust. 2013; 41(1): 65–72.

[20] Cochran WG. Sampling Techniques (3rd edition), New York, USA, Wiley; 1977.

[21] Stevens DL, Olsen AR. Spatially balanced sampling of natural resources. J Am Stat Assoc. 2004; 99: 262–278.

[22] Stevens DL, Olsen AR. Variance estimation for spatially balanced samples of environmental resources. Environmetrics. 2003; 14: 593–610.

[23] Wardrop D, Kentula M, Stevens D, Jensen S, Brooks R. Assessment of wetland condition: An example from the Upper Juniata watershed in Pennsylvania. USA Wetlands. 2007; 27: 416–431.

[24] Fancy S, Gross J, Carter S. Monitoring the condition of natural resources in US national parks. Environ Monit and Assess. 2009; 151: 161–174. 10.1007/s10661-008-0257-y 18509737

[25] Hasler M, Hornik K. TSP: Traveling Salesperson Problem (TSP). R package version 1.0–9. 2014. Available: http://CRAN.R-project.org/package=TSP.

[26] Kincaid TM, Olsen AR. Spsurvey: Spatial Survey Design and Analysis. R package version 2.5. 2012. Available: www.epa.gov.au/nheerl/arm/.

[27] R Core Team. R: A language and environment for statistical computing R Foundation for Statistical Computing, Vienna, Austria 2012 Available: http://www.R-project.org/

[28] Stevens DL. Variable density grid-based sampling designs for continuous spatial populations. Environmetrics. 1997; 8,:167–195.

[29] Seager J. Transect Measure. 2014. Available: http://www.seagis.com.au

[30] CATAMI Technical Working Group, 2013. CATAMI classification scheme for scoring marine biota and substrata in underwater imagery—Technical Report. Available: http://catami.org/classification. Version 1.3

[31] Gavrilov AN, Siwabessy PJW, Parnum IM. Multibeam echo sounder backscatter analysis. Curtin University of Technology. 2005

[32] Daniell J, Jorgensen DC, Anderson T, Borissova I, Burq S, Heap AD, et al. & Shipboard Party. Frontier Basins of the West Australian Continental Margin: Post‐survey Report of Marine Reconnaissance and Geological Sampling Survey GA2476. Geoscience Australia Record 2009/38, Canberra. 2010.

[33] Lucieer VL. Object-oriented classification of sidescan sonar data for mapping benthic marine habitats. Int J Remote Sens. 2008; 29(3): 905–921.

[34] Lucieer VL, Stein A, Fisher P. Multivariate texture based segmentation of remotely sensed imagery for extraction of objects and their uncertainty. Int J Remote Sens. 2005; 26(14): 2917–2936.

[35] Lucieer V, Hill NA, Barrett NS, Nichol S . Do marine substrates ‘look’ and ‘sound’ the same? Supervised classification of multibeam acoustic data using autonomous underwater vehicle images. Estuar Coast Shelf Sci. 2013; 117: 94–106.

[36] Congalton RG. A review of assessing the accuracy of classifications of remotely sensed data. Remote Sens Environ. 1991; 37: 35–46.

[37] Lillesand TM, Kiefer RW. Remote Sensing and Image Interpretation. (Third edition) John Wiley and Sons Inc, New York; 1994.

[38] Altman DG. Practical statistics for medical research London, England, Chapman & Hall; 1991.

[39] Huang Z, Siwabessy J, Nichol SL, Brooke BP. Predictive mapping of seabed substrata using high-resolution multibeam sonar data: A case study from a shelf with complex geomorphology. Mar Geol. 2014; 357: 37–52.

[40] Barrett N, Seiler J, Anderson T, Williams S, Nichol S, Hill N. Autonomous underwater Vehicle (AUV) for mapping marine biodiversity in coastal and shelf waters: Implications for marine management. Proceedings OCEANS’10 IEEE Conference, Sydney, 24–27 May 2010.

[41] Hill NA, Barrett NB, Lawrence E, Hulls J, Dambacher JM, Nichol S, et al What's in a reserve? Quantifying fish assemblages in a large offshore marine reserve using spatially-balanced and non-extractive sampling. PLoS One. 2014; 9(10). 10.1371/journal.pone.0110831 PMC4215995 25360763

[42] Rao JNK, Graham JE. Rotation designs for sampling on repeated occasions. J Am Stat Assoc. 1964; 59: 492–509.

