# Supplementary figures and images for: Mapping Habitats and Developing Baselines in Offshore Marine Reserves with Little Prior Knowledge: A Critical Evaluation of a New Approach
Source: PLoS One. 2015 Oct 23;10(10):e0141051. doi: 10.1371/journal.pone.0141051 (PMC4619713; doi:10.1371/journal.pone.0141051)

**S1A Figure**

**
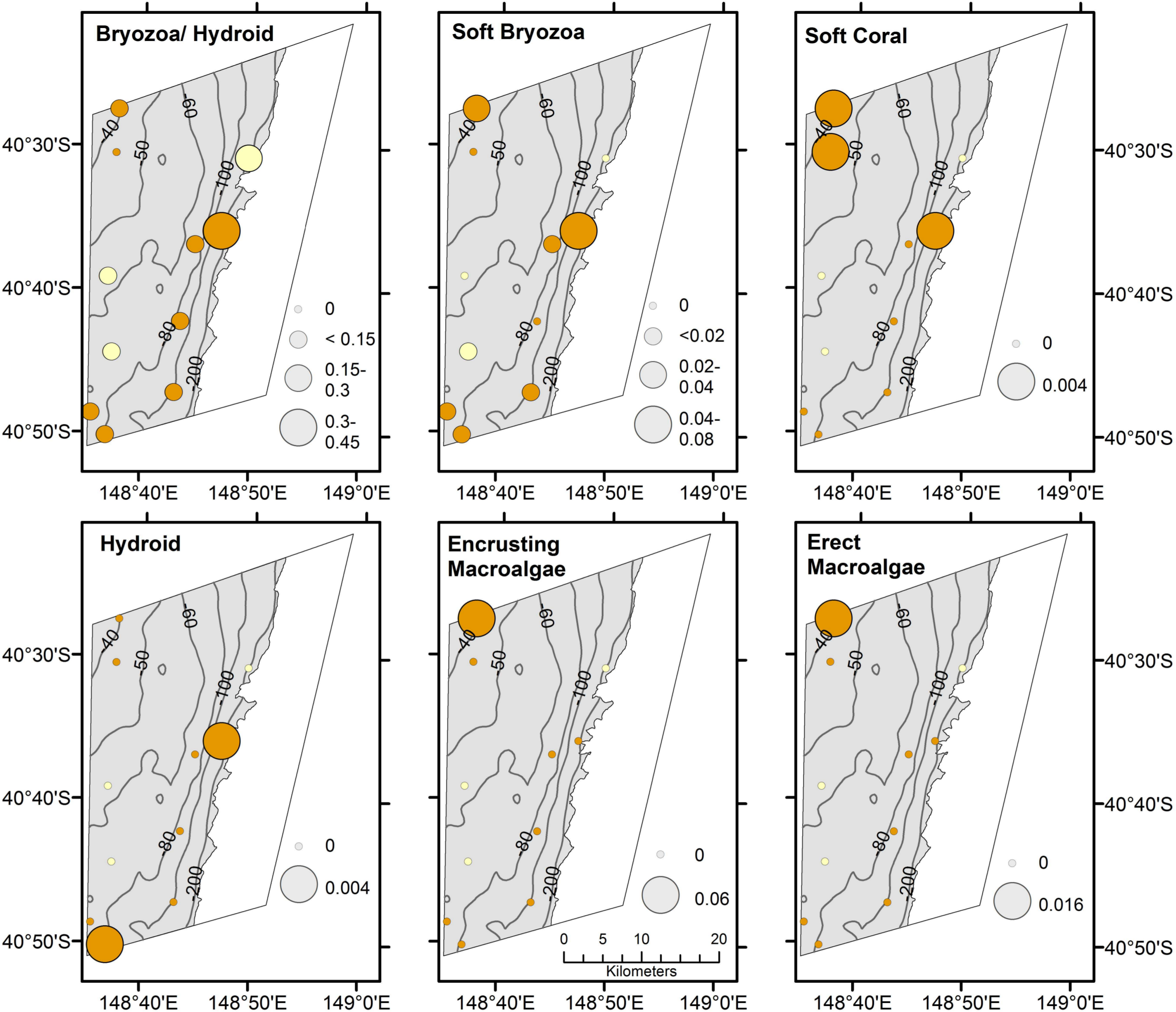
**

**S1B Figure**

**
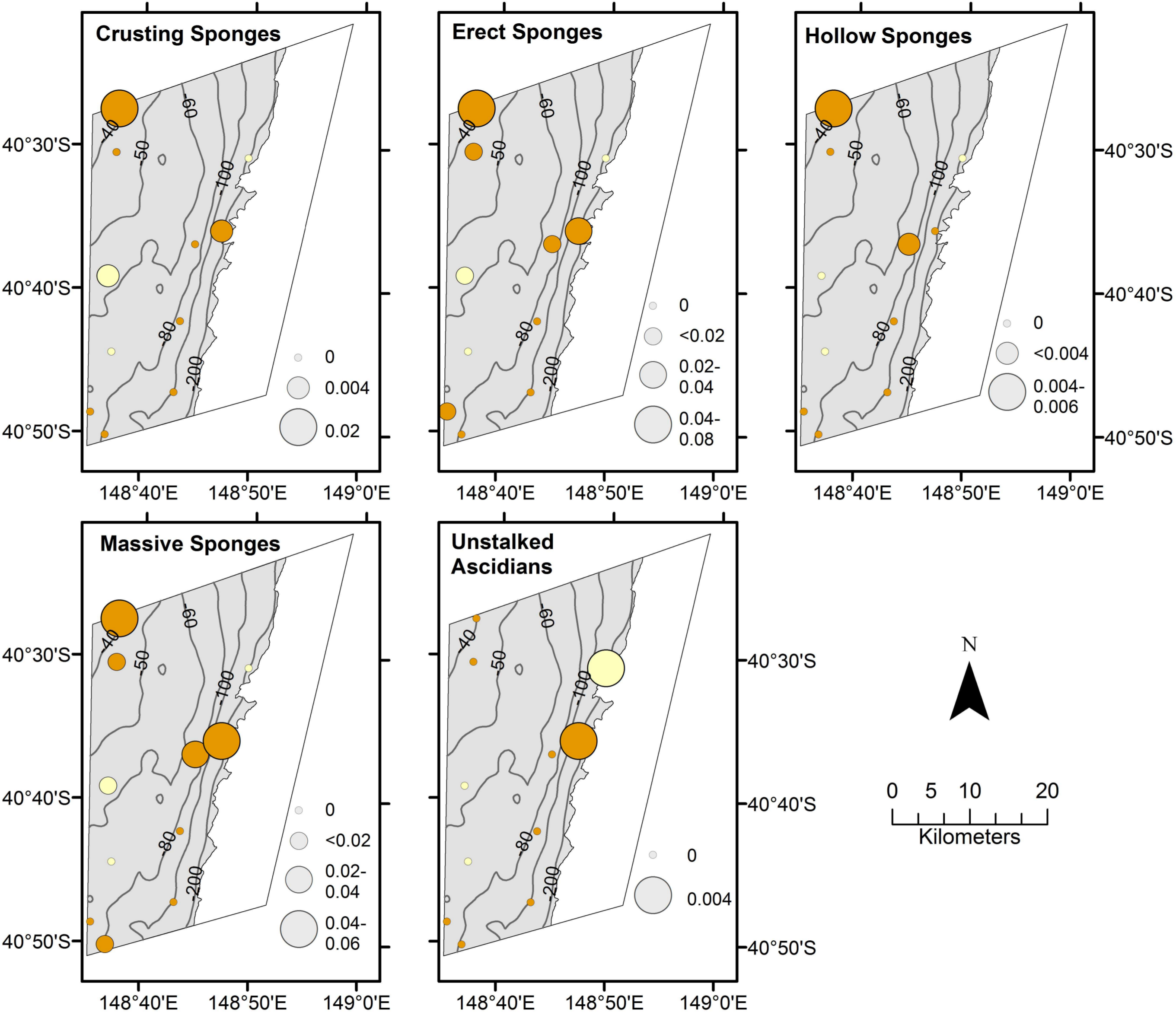
**

Supplement: S1 Fig — Orange bubbles represent mixed habitat and yellow represent soft habitat. (DOCX) [file pone.0141051.s004.docx]
