# Supplementary material for: Mapping Habitats and Developing Baselines in Offshore Marine Reserves with Little Prior Knowledge: A Critical Evaluation of a New Approach
Source: PLoS One. 2015 Oct 23;10(10):e0141051. doi: 10.1371/journal.pone.0141051 (PMC4619713; doi:10.1371/journal.pone.0141051)
Supplement: S1 Table — (DOCX) [file pone.0141051.s005.docx]

**S1 Table 1**

| **Dominant Substratum Type** | **Dominant Substrate Cover** | **Dominant Substratum Modifier** | **Subdominant Substratum Type** | **Subdominant Substrate Cover** | **Subominant Substratum Modifier** | **MBES Classification** |
| --- | --- | --- | --- | --- | --- | --- |
| Consolidated\|Rock\| | 81-100 | Veneer | Unconsolidated\|Sand/Mud\| | 1-20 |  | Hard |
| Consolidated\|Rock\| | 81-100 |  | Unconsolidated\|Sand/Mud\| | 1-20 | Veneer | Hard |
| Consolidated\|Rock\| | 81-100 |  |  |  |  | Hard |
| Consolidated\|Rock\| | 61-80 |  | Unconsolidated\|Sand/Mud\| | 21-40 | Veneer | Hard |
| Consolidated\|Rock\| | 61-80 |  | Unconsolidated\|Sand/Mud\| | 21-40 |  | Hard |
| Consolidated\|Rock\| | 41-60 | Veneer | Unconsolidated\|Sand/Mud\| | 41-60 |  | Mixed |
| Consolidated\|Rock\| | 41-60 |  | Unconsolidated\|Sand/Mud\| | 41-60 |  | Mixed |
| Unconsolidated\|Sand/Mud\| | 81-100 | Veneer | Unconsolidated\|Pebble/Gravel\|Pebble | 1-20 |  | Mixed |
| Unconsolidated\|Sand/Mud\| | 81-100 | Veneer | Unconsolidated\|Pebble/Gravel\|Pebble | 21-40 |  | Mixed |
| Unconsolidated\|Sand/Mud\| | 81-100 | Veneer | Consolidated\|Rock\| | 1-20 |  | Mixed |
| Unconsolidated\|Sand/Mud\| | 81-100 | Veneer |  |  |  | Mixed |
| Unconsolidated\|Sand/Mud\| | 61-80 | Veneer | Unconsolidated\|Sand/Mud\| | 21-40 | Veneer | Mixed |
| Unconsolidated\|Sand/Mud\| | 61-80 | Veneer | Unconsolidated\|Sand/Mud\| | 21-40 |  | Mixed |
| Unconsolidated\|Sand/Mud\| | 61-80 | Veneer | Consolidated\|Rock\| | 21-40 |  | Mixed |
| Unconsolidated\|Sand/Mud\| | 41-60 | Veneer | Unconsolidated\|Sand/Mud\| | 21-40 |  | Mixed |
| Unconsolidated\|Sand/Mud\| | 41-60 | Veneer | Unconsolidated\|Sand/Mud\| | 41-60 |  | Mixed |
| Unconsolidated\|Sand/Mud\| | 41-60 | Veneer | Consolidated\|Rock\| | 41-60 |  | Mixed |
| Unconsolidated\|Sand/Mud\| | 41-60 |  | Consolidated\|Rock\| | 41-60 |  | Mixed |
| Consolidated\|Rock\| | 41-60 |  | Unconsolidated\|Sand/Mud\| | 21-40 | Veneer | Mixed |
| Consolidated\|Rock\| | 41-60 |  | Unconsolidated\|Sand/Mud\| | 41-60 | Veneer | Mixed |
| Unconsolidated\|Sand/Mud\| | 81-100 |  | Unconsolidated\|Sand/Mud\| | 1-20 | Veneer | Mixed |
| Unconsolidated\|Sand/Mud\| | 81-100 |  | Consolidated\|Rock\| | 1-20 |  | Mixed |
| Unconsolidated\|Sand/Mud\| | 61-80 |  | Unconsolidated\|Sand/Mud\| | 21-40 | Veneer | Mixed |
| Unconsolidated\|Sand/Mud\| | 61-80 |  | Consolidated\|Rock\| | 21-40 |  | Mixed |
| Unconsolidated\|Sand/Mud\| | 41-60 |  | Unconsolidated\|Sand/Mud\| | 21-40 | Veneer | Mixed |
| Unconsolidated\|Sand/Mud\| | 41-60 |  | Unconsolidated\|Sand/Mud\| | 41-60 | Veneer | Mixed |
| Unconsolidated\|Sand/Mud\| | 41-60 |  | Unconsolidated\|Sand/Mud\| | 61-80 | Veneer | Mixed |
| Unconsolidated\|Sand/Mud\| | 41-60 |  | Consolidated\|Cobble\| | 41-60 |  | Mixed |
| Unconsolidated\|Pebble/Gravel\|Pebble | 61-80 |  | Unconsolidated\|Sand/Mud\| | 21-40 |  | Soft |
| Unconsolidated\|Sand/Mud\| | 81-100 |  | Unconsolidated\|Sand/Mud\| | 1-20 | Bryozoa Crust | Soft |
| Unconsolidated\|Sand/Mud\| | 81-100 |  | Unconsolidated\|Pebble/Gravel\|Pebble | 1-20 |  | Soft |
| Unconsolidated\|Sand/Mud\| | 81-100 |  | Unconsolidated\|Pebble/Gravel\|Pebble | 21-40 |  | Soft |
| Unconsolidated\|Sand/Mud\| | 81-100 |  | Unconsolidated\|Pebble/Gravel\|Biogenic\| Screwshells | 1-20 |  | Soft |
| Unconsolidated\|Sand/Mud\| | 81-100 |  |  | 21-40 |  | Soft |
| Unconsolidated\|Sand/Mud\| | 81-100 |  |  |  |  | Soft |
| Unconsolidated\|Sand/Mud\| | 61-80 |  | Unconsolidated\|Sand/Mud\| | 21-40 | Bryozoa Crust | Soft |
| Unconsolidated\|Sand/Mud\| | 61-80 |  | Unconsolidated\|Pebble/Gravel\|Pebble | 21-40 |  | Soft |
| Unconsolidated\|Sand/Mud\| | 61-80 |  |  |  |  | Soft |
| Unconsolidated\|Sand/Mud\| | 41-60 |  | Unconsolidated\|Pebble/Gravel\|Pebble | 41-60 |  | Soft |
| Unconsolidated\|Sand/Mud\| | 81-100 | Bryozoa Crust | Unconsolidated\|Sand/Mud\| | 41-60 |  | Soft |
| Unconsolidated\|Sand/Mud\| | 41-60 | Bryozoa Crust | Unconsolidated\|Sand/Mud\| | 41-60 |  | Soft |
| Unconsolidated\|Sand/Mud\| | 41-60 |  | Unconsolidated\|Sand/Mud\| | 41-60 | Bryozoa Crust | Soft |
