# Supplementary material for: Mapping Habitats and Developing Baselines in Offshore Marine Reserves with Little Prior Knowledge: A Critical Evaluation of a New Approach
Source: PLoS One. 2015 Oct 23;10(10):e0141051. doi: 10.1371/journal.pone.0141051 (PMC4619713; doi:10.1371/journal.pone.0141051)
Supplement: S2 Table — (DOCX) [file pone.0141051.s006.docx]

**S2A Table. Accuracy assessment of the hand digitised classification of multibeam and AUV classified points.**

| **Digitised** |  |  |  |  |  |
| --- | --- | --- | --- | --- | --- |
|  | Hard | Mixed | Soft | Classification Overall | |
| Hard | 81 | 23 | 64 | 168 | 48.21% |
| Mixed | 36 | 5 | 15 | 56 | 8.93% |
| Soft | 58 | 37 | 401 | 496 | 80.85% |
| Truth Overall | 175 | 65 | 480 | 720 |  |
| User Accuracy (Recall) | 46.29% | 7.69% | 83.54% |  |  |
|  |  |  |  |  |  |
| Overall Accuracy (OA) | 67.64% |  |  |  |  |
| Kappa | 0.322 |  |  |  |  |

**S2B Table.** **Accuracy assessment of the GEOBIA classification of multibeam and AUV classified points.**

| **GEOBIA** |  |  |  |  |  |
| --- | --- | --- | --- | --- | --- |
|  | Hard | Mixed | Soft | Classification Overall | |
| Hard | 84 | 49 | 35 | 168 | 48.21% |
| Mixed | 29 | 11 | 16 | 56 | 8.93% |
| Soft | 59 | 77 | 360 | 496 | 80.85% |
| Truth Overall | 172 | 137 | 411 | 720 |  |
| User Accuracy (Recall) | 48.84% | 8.03% | 87.59% |  |  |
|  |  |  |  |  |  |
| Overall Accuracy (OA) | 63.19% |  |  |  |  |
| Kappa | 0.314 |  |  |  |  |

**S2C Table. Accuracy assessment of the ARC classification of multibeam and AUV classified points.**

| **ARC** |  |  |  |  |  |
| --- | --- | --- | --- | --- | --- |
|  | Hard | Mixed | Soft | Classification Overall | |
| Hard | 0 | 2 | 2 | 4 | 0.00% |
| Mixed | 26 | 51 | 45 | 122 | 41.80% |
| Soft | 17 | 64 | 413 | 494 | 83.60% |
| Truth Overall | 43 | 117 | 460 | 620 |  |
| User Accuracy (Recall) | 0.00% | 43.59% | 89.78% |  |  |
|  |  |  |  |  |  |
| Overall Accuracy (OA) | 74.84% |  |  |  |  |
| Kappa | 0.322 |  |  |  |  |
